# Spotlight On Early Career Researchers: an interview with Lovorka Stojic

**DOI:** 10.1038/s42003-019-0466-7

**Published:** 2019-06-14

**Keywords:** Long non-coding RNAs, Cancer, Careers, Lab life

## Abstract

Dr. Lovorka Stojic is a postdoctoral research fellow at the University of Cambridge and will start her own research group at the Barts Cancer Institute this fall. Her research focuses on understanding how long noncoding RNAs and RNA-binding proteins regulate key cellular processes and how dysregulation of these processes can contribute to human diseases such as cancer. As part of our series on early-career researchers, we asked Dr. Stojic to tell us about her research and career path. She also shares her challenges from juggling between multiple roles and advice for job applications.

**Figure:**
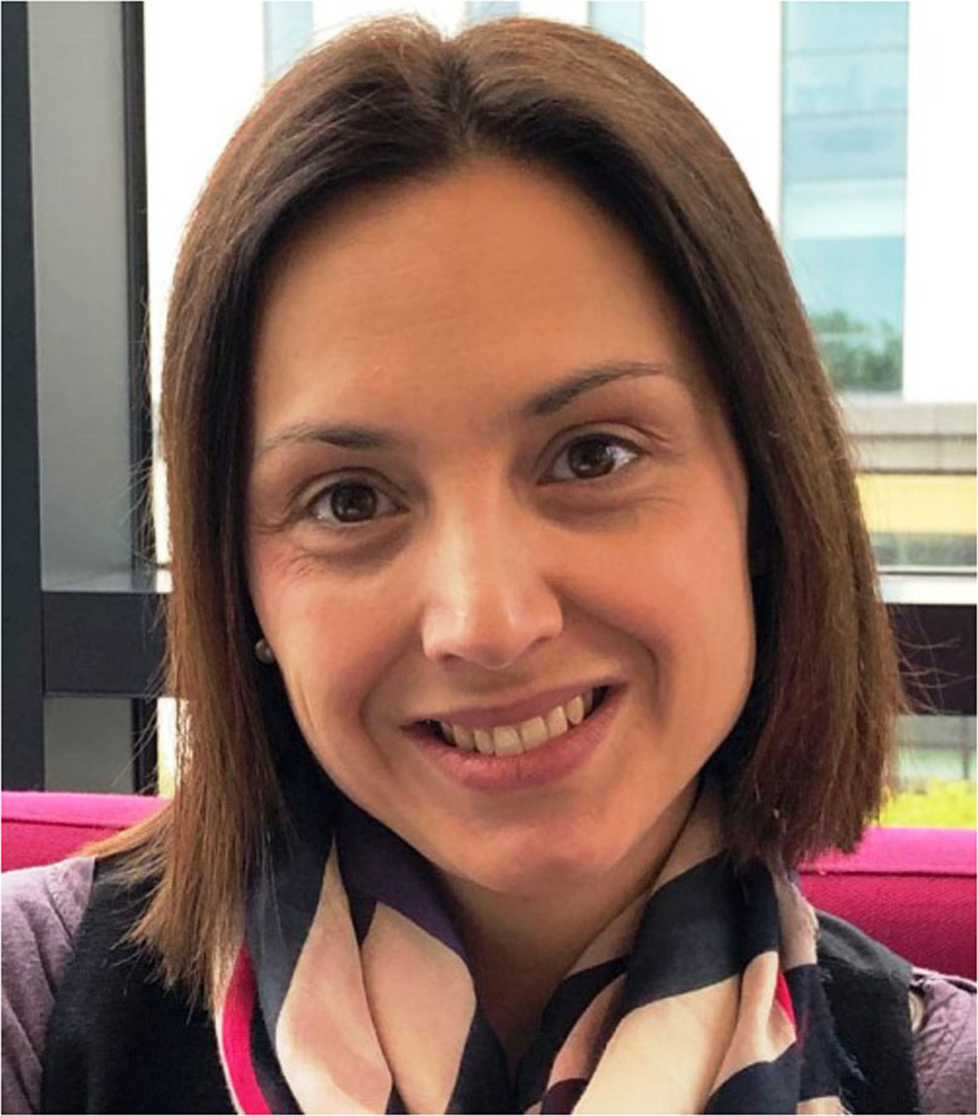
Image credit: Lovorka Stojic

Please tell us about your research interests.

I am interested in how RNA-mediated mechanisms, such as those mediated by long noncoding RNAs (lncRNAs) and RNA binding proteins (RBP), regulate fundamental cellular processes. My current research focuses on the regulatory principles underlying the role of lncRNAs in cell division, and how lncRNA regulates genome instability. To understand the function and biological relevance of lncRNAs in the maintenance of genome stability, I use various approaches from genomics, proteomics, imaging and functional cell biology. Interestingly, many lncRNAs and RBPs are dysregulated in human diseases. One of my mid-term goals is to understand how defects in RNA regulatory networks contribute to cancer. lncRNA biology really intrigues me as they have potential to regulate gene expression at so many levels. Working with lncRNAs often feels like solving Agatha Christie crime mysteries, which requires great scientific skills together with an element of pure intuition.

What has your journey been to this point?

After finishing my undergraduate degree in molecular biology at the University of Zagreb in Croatia, I decided to do my PhD at the University of Zurich in the laboratory of Prof. Josef Jiricny. There I investigated how DNA repair proteins activate DNA damage checkpoints to maintain genome stability. At the end of my PhD, I became fascinated by epigenetics and decided to join Dr. Valerio Orlando’s laboratory in Italy to do my first postdoc. In his laboratory, I was introduced to key chromatin-mediated mechanisms that control gene expression. I then decided to move to Cambridge (UK), where I started my second postdoc in the group of Dr. Adele Murrell at the University of Cambridge. Her laboratory mainly focuses on imprinting and chromatin structure. When I joined her lab, they had just identified a new lncRNA. LncRNAs were being discovered rapidly at the time, many of them acting as binding partners of Polycomb group proteins. That was the link between my previous work and the unknown world of lncRNAs. I will never forget the first day I joined the Murrell lab, when she told me: “We have this cool noncoding RNA and nobody knows what it does: it might bind Polycomb; it might regulate imprinting. Who knows? Go and figure it out!”

I realised very quickly that if I wanted to study these lncRNAs, l had to become familiar with key techniques. I had the fortune to receive a CRUK Travel Award, which allowed me to visit one of the leading labs in lncRNA biology. Being a visiting scientist in Dr. John Rinn’s lab (then at Harvard University) was a great experience, both professionally and personally. After my return to Cambridge from Boston, Adele’s laboratory relocated to the University of Bath unexpectedly. Moving again was not an option for me, also for family reasons, so I decided to stay in Cambridge, where I took advantage of the supervision of two senior group leaders, Dr. Fanni Gergely and Dr. Duncan Odom. Both Fanni and Duncan gave me the freedom to develop my own research on the role of lncRNAs for regulation of cell division. I realised that the combination of functional cell biology and genomics serves as a powerful tool to study lncRNAs and that the expertise of the two laboratories was really instrumental for me. My postdoctoral journey was probably unusual as I had four different supervisors from completely different fields, which all shaped me in a unique way as a scientist. It will definitely help me establish my own research group at the Barts Cancer Institute in London as of this September.

What are your predictions for your field in the near future?

Although lncRNAs were first discovered more than 20 years ago, we are still learning how these RNAs operate, simply because for many years we lacked the right experimental tools. With the development of new biochemical methods, we are now in a better position to decipher structural and molecular functions of lncRNAs. However, it is challenging that some of these newly identified lncRNAs may not be functional. Thus, we need to stay vigilant when studying novel lncRNAs.

I see the lncRNA field moving in three directions in the foreseeable future. First, given that a large proportion of the genome codes for lncRNAs and that they are functionally versatile, it is not surprising that they are often dysregulated in cancer. One of my future goals is to understand how lncRNAs functionally contribute to known hallmarks of cancer. Till now most of the work on the role of lncRNAs in cancer biology has been done in cancer cell lines, but the field is shifting towards mouse models and organoids. Organoids are a good compromise between cell lines and living animals and can be useful especially for scientists working on human-specific lncRNAs. We can now engineer organoids and even model human tumorigenesis in vitro. Furthermore, patient-derived tumor organoids and/or patient-derived xenografts serve as good models to test whether lncRNAs have a clinical potential. Organoids are suitable for live-cell imaging, which allows one to study dynamic processes in real time. This brings me to my second direction, which I am personally very excited about, RNA imaging. With the development of new tools used to image RNA at the single-molecule level, we can now track lncRNAs in living cells, and ask so many questions about their role in cell cycle progression. Third direction is epitranscriptomics. We know very little about dynamics and functions of different RNA modifications on lncRNAs and how these RNA modifications affect their structure, stability, localisation as well as cellular processes they regulate. With the development of new high-throughput mapping approaches and functional studies, we will learn more about the importance of RNA modifications and their contribution to different cellular processes and diseases.

Can you speak of any challenges that you have overcome?

It was challenging for me to go back to the lab after both of my maternity breaks during my postdoctoral training in Cambridge, and to coordinate my work with parenting. I often found myself reading papers while waiting for my kids’ football games or swimming to end. My personal challenge is trying to be a good mother, partner, scientist, and mentor at the same time. Another scientific challenge, which nobody prepares me for, is the transition from a postdoc to a group leader position. As someone who will soon start an independent research group, I can only say that you need to be persistent, resilient, and have a thick skin. Every unsuccessful interview presents an opportunity to learn more for a better interview, until you are ready for the right job. It takes time to master your job application skills, which means that you have to take the best out of rejections and ask for feedback after each interview.

What advice would you give to your younger self?

Follow what your science leads you to and stay enthusiastic about your research. At the same time, take criticism from your peers, colleagues, and collaborators. Don’t be afraid to reach out for help if needed, and start to network as early as you can. You never know when you will need it.

What is your favourite RNA?

Any long noncoding RNA that is functional.

*This interview was conducted by Associate Editor Jung-Eun Lee*.

